# Analysis of OXA-204 carbapenemase-producing *Enterobacteriaceae* reveals possible endoscopy-associated transmission, France, 2012 to 2014

**DOI:** 10.2807/1560-7917.ES.2017.22.49.17-00048

**Published:** 2017-12-07

**Authors:** Anaïs Potron, Sandrine Bernabeu, Gaëlle Cuzon, Valérie Pontiès, Hervé Blanchard, Elise Seringe, Thierry Naas, Patrice Nordmann, Laurent Dortet

**Affiliations:** 1 National Reference Centre for Antibiotic Resistance, (division of carbapenemase-producing Enterobacteriaceae), Le Kremlin-Bicêtre, France; 2 Department of Bacteriology, University Hospital of Besançon, Université of Franche-Comté, Besançon, France; 3 Bacteriology-Hygiene Unit, Assistance Publique/Hôpitaux de Paris, Bicêtre Hospital, Le Kremlin-Bicêtre, France; 4 EA7361 “Structure, Dynamic, Function and Expression of Broad Spectrum beta-Lactamases”, Paris-Sud University, LabEx Lermit, Faculty of Medicine, Le Kremlin-Bicêtre, France; 5 Joint Research Unit EERA “Evolution and Ecology of Resistance to Antibiotics,” Institut Pasteur-APHP-Université Paris-Sud, Paris, France; 6 Santé Publique France, The French Public Health Agency, Saint-Maurice, France; 7 Regional Coordinating Centre for Nosocomial Infection Control (C-CLIN Paris Nord), Paris, France.; 8 Emerging Antibiotic Resistance Unit, Medical and Molecular Microbiology, Department of Medicine, University of Fribourg, Fribourg, Switzerland; 9 Institut National de la Santé et de la Recherche Médicale (INSERM) European Unit (LEA Paris, IAME, France), University of Fribourg, Switzerland; 10 National Reference Centre for Emerging Antibiotic Resistance, Fribourg, Switzerland; 11 Institute for Microbiology, University hospital and University of Lausanne, Lausanne, Switzerland

**Keywords:** carbapenems, outbreak, OXA-48-like, antibiotic resistance, endoscope, nosocomial

## Abstract

OXA-48-like beta-lactamase producing bacteria are now endemic in several European and Mediterranean countries. Among this carbapenemase family, the OXA-48 and OXA-181 variants predominate, whereas other variants such as OXA-204 are rarely reported. Here, we report the molecular epidemiology of a collection of OXA-204-positive enterobacterial isolates (n = 29) recovered in France between October 2012 and May 2014. This study describes the first outbreak of OXA-204-producing *Enterobacteriaceae* in Europe, involving 12 isolates of an ST90 *Escherichia coli* clone and nine isolates of an ST147 *Klebsiella pneumoniae* clone. All isolates co-produced the cephalosporinase CMY-4, and 60% of them co-produced the extended-spectrum beta-lactamase CTX-M-15. The *bla*_OXA-204_ gene was located on a 150-kb IncA/C plasmid, isolated from various enterobacterial species in the same patient, indicating a high conjugative ability of this genetic vehicle.

## Introduction

Since the 2000s, the carbapenem-hydrolysing beta-lactamase OXA-48 has rapidly and widely disseminated and is now endemic in several European and Mediterranean countries [1-4]. Since its discovery, eleven variants of OXA-48 have been reported, classified into two main groups [1,5-13]. The first group contains variants with significant carbapenemase activity, such as OXA-48, OXA-162, OXA-181 or OXA-204 [5-7]. Some variants, such as OXA-232 and OXA-244, possess a hydrolytic profile similar to that of OXA-48 but have a lower capacity to hydrolyse imipenem and temocillin [8-10]. The second group of OXA-48-like variants includes beta-lactamases with extended-spectrum hydrolysis properties and without any significant carbapenemase activity because of deletions in the active site of the enzyme, such as OXA-163, OXA-247, or OXA-405 [11-13]. In most of these cases, the *bla*_OXA-48_-like genes are plasmid-borne and are associated with insertion sequences involved in their mobilisation and expression [1,7,9].

OXA-204 was first identified in 2012 in a *Klebsiella pneumoniae* isolate from Tunisia [7]. In this strain, the *bla*_OXA-204_ was co-located with a *bla*_CMY-4_ gene on a conjugative IncA/C-type plasmid [7]. The *bla*_OXA-204_ gene was part of a transposon Tn*2016* that consisted of one copy of insertion sequence (IS) IS*Ecp1*, disrupted by an IS*Kpn15* element, and a truncated *lysR* transcriptional regulator [7]. Since that report, OXA-204 has only been identified in two other *K. pneumoniae* isolates and a single *Escherichia coli* isolate, all recovered in Tunisia [14,15]. In those two strains, the *bla*_OXA-204_ gene was associated with the IS*Ecp1* element, in one of the two cases truncated by another IS element [14,15].

Our study aimed to compare the genetic features of OXA-204 beta-lactamase-producing strains recovered in France by analysing a collection of 29 *bla*_OXA-204_-positive enterobacterial isolates recovered from October 2012 to May 2014. The genetic context and the location of the *bla*_OXA-204_ gene were investigated. Finally, a clonal relationship analysis allowed us to identify a regional outbreak in France possibly related to an endoscope.

## Methods

### Bacterial isolates

We investigated a total of 29 OXA-204 beta-lactamase-producing enterobacterial isolates. All isolates had been recovered from clinical specimens and had been received between October 2012 and May 2014 at the National Reference Centre (NRC) for Antibiotic Resistance (division of carbapenemase-producing *Enterobacteriaceae*), France. The distribution of clinical samples was as follows: 12 rectal swabs, 12 urine samples, four bile samples and one pus specimen. Isolates were identified using matrix-assisted laser desorption ionization time-of-flight (MALDI-TOF) mass-spectrometry (Maldi Biotyper, Bruker Daltonics, France).

### Susceptibility testing

Antimicrobial susceptibilities were determined by disk diffusion method on Mueller-Hinton agar (Bio-Rad, Marnes-la-Coquette, France) and interpreted according to the European Committee on Antimicrobial Susceptibility Testing (EUCAST) guidelines [16]. In addition, minimal inhibitory concentrations (MICs) were determined for carbapenems (imipenem, meropenem, ertapenem) and tigecycline using E-tests (bioMérieux, Marcy-l’Etoile, France), and for colistin using broth microdilution according to EUCAST recommendations.

### PCR and sequencing of beta-lactamase-encoding genes

Whole-cell DNA was extracted using the QiaAmp DNA minikit (Qiagen, Courtaboeuf, France). All isolates were screened by PCR for the Ambler class A, B and D carbapenemase-encoding genes *bla*_KPC_, *bla*_IMP_, *bla*_VIM_, *bla*_NDM_ and *bla*_OXA-48-like_ as previously described [6,17,18]. Detection of other beta-lactamase genes such as *bla*_CTX-M_ and *bla*_AmpC-like_ was performed with internal primers, as described previously [17]. PCR products were analysed on agarose gel. In case of positive signal, the full-length genes (basically *bla*_CTX-M_ and *bla*_CMY_) were amplified and sequenced by using the amplification primers with an automated sequencer (ABI PRISM 3100; Applied Biosystems) as previously described [17]. The nucleotide and deduced protein sequences were analysed with software from the National Center for Biotechnology Information (www.ncbi.nlm.nih.gov).

### Strain typing

Multilocus sequence typing (MLST) with seven housekeeping genes (*rpoB*, *gapA*, *mdh*, *pgi*, *phoE*, *infB* and *tonB*) was performed for *K. pneumoniae* isolates according to Diancourt et al. [19]. Allele sequences and sequence types (STs) were verified at the Institut Pasteur’s whole genome MLST database [20]. Fragments of seven housekeeping genes (*adk*, *fumC*, *gyrB*, *icd*, *mdh*, *purA* and *recA*) were amplified and sequenced for *E. coli* isolates as described on EnteroBase [21]. A different allele number was given to each distinct sequence within a locus, and a distinct ST number was attributed to each distinct combination of alleles.

### Clonality analysis using repetitive element palindromic PCR (rep-PCR)

To evaluate their clonal relationship, all *E. coli* and *K. pneumoniae* isolates were subjected to Diversilab, a semi-automated rep-PCR (bioMérieux, Marcy-L’Etoile, France). As recommended by the manufacturer, a cut-off for similarity of 95% defined a cluster.

### Plasmid DNA analysis and mating-out assays

Plasmid DNAs were extracted using the Kieser method [22], and analysed by agarose gel electrophoresis using the *E. coli* NCTC50192 strain that harbours four plasmids of 154, 66, 48 and 7 kb as plasmid size marker. Direct transfer of the carbapenem resistance markers was attempted by liquid mating-out assays at 37 °C using sodium azide-resistant *E. coli* J53 as recipient, as previously described [23]. Selection was performed on agar plates supplemented with ertapenem (0.5 µg/mL) and sodium azide (100 µg/mL).

### Replicon and transposon typing

PCR-based replicon typing of the main plasmid incompatibility groups reported in *Enterobacteriaceae* was performed as previously described [24]. Genetic structures surrounding the *bla*_OXA-204_ gene were determined using the primers listed in Table 1.

**Table 1:** Primers used for Tn*2016* PCR mapping

Primer	Sequence (5’ to 3’)	PCR product size (bp)
IS*Ecp*1A	TGCAGGTCTTTTTCTGCTCC	1,099
IS*Kpn*15–5’ext	CTGCGTGGCTATGTGCTCTG	
IS*Kpn*15-for	GGTGTTCGGTGACGAGATTAGC	1,955
OXA-48–5’ext	ATTCCAGAGCACAACTACGC	
IS*Ecp*1P +	TGCTCTGTGGATAACTTGCA	998
OXA48B	GAGCACTTCTTTTGTGATGGC	

## Results

### Bacterial isolates

A total of 29 OXA-204-producing enterobacterial isolates were received at the NRC for Antibiotic Resistance from October 2012 to May 2014. These isolates were sent to the NRC because they exhibited decreased susceptibility to carbapenems and/or because they were isolated from a patient who was identified in epidemiological investigations around an infected or colonised patient. These isolates included 11 *K. pneumoniae*, 15 *E. coli*, one *Proteus mirabilis,* one *Citrobacter freundii* and one *Serratia marcescens* (Table 2). The 29 strains were isolated from 24 patients. Of these 29 OXA-204 producers, 27 were isolated from 22 patients (n = 22) located in the same geographical area (Paris area, Ile-de-France).

**Table 2:** Phenotypic and genetic features associated with OXA-204-beta-lactamase producing *Enterobacteriaceae*, France, 2012–14 (n = 29)

Species /clone	Number of isolates	Sequence type	Beta-lactams MIC range (µg/mL)			Genetic location of blaOXA-204	Incompatibility group of blaOXA-204-carrying plasmid	Non-beta-lactam-associated resistance (number of strains)	Associated broad-spectrum beta-lactamasesa (number of strains)	Close genetic environment of the blaOXA-204 gene
			ERT	IMP	MER					
*Escherichia coli *										
A	12	ST90	0.38–32	0.19–12	0.094–6	Plasmid	IncA/C	Gm (12), Tm (12), Q (12), Tet(12), Sxt (10)	CMY-4 (12), CTX-M-15 (9)	Tn*2016*
B	1	ST104	0.5	0.38	0.25	Plasmid	IncA/C	Tet	CMY-4	IS*Ecp1*
C	1	ST617	1	0.25	0.25	Plasmid	IncA/C	Gm, Tm, Q, Tet, Sxt	CMY-4	Tn*2016*
D	1	ST949	0.75	0.5	0.19	Plasmid	IncA/C	Q, Tet, Sxt	CMY-4	IS*Ecp1*
*Klebsiella pneumoniae *										
A	9	ST147	3– >32	1–12	1– >32	Plasmid	IncA/C	Ak (1), Tm (8), Gm (6), Q (9), Tet (9), Sxt (8), Fos (7)	CMY-4 (9), CTX-M-15 (9)	IS*Ecp1*
B	1	ST1683^b^	3	0.25	0.19	Plasmid	IncA/C	Gm, Tm, Tet	CMY-4	Tn*2016*
C	1	ST1709^b^	1	0.25	0.12	Plasmid	IncA/C	Gm, Tm, Tet, Tig	CMY-4	Tn*2016*
*Proteus mirabilis*	1	ND	1.5	0.38	0.094	Plasmid	IncA/C	Gm, Tm, Sxt	CMY-4	IS*Ecp1*
Citrobacter freundii	1	ND	3	1	0.5	Plasmid	IncA/C	Gm, Tm, Q, Tet, Tig	CMY-4	Tn2016
Serratia marcescens	1	ND	0.75	0.75	0.5	Plasmid	IncA/C	Gm, Tm, Tet, Tig	CMY-4	Tn2016

Ak: amikacin; ERT: ertapenem; Fos: fosfomycin; Gm: gentamicin; IMP: imipenem; MER: meropenem; MIC: minimum inhibitory concentration; ND: not determinable; Q: quinolones; Sxt: sulfamethoxazole-trimethoprim; Tet: tetracycline; Tig: tigecycline; Tm: tobramycin.

^a^ Resistance markers co-harboured on the *bla*_OXA-204_-carrying plasmid are underlined.

^b^ ST1683 ad ST1709 are new sequence types.

### Susceptibility to beta-lactams and related beta-lactamase genes

According to the EUCAST guidelines, 21 isolates were susceptible to imipenem and meropenem. Of the 15 *E. coli* isolates, 14 were susceptible to imipenem and meropenem whereas only four of the 11 *K. pneumoniae* isolates were susceptible to those antibiotics (Table 2). By contrast, 21 of the 29 OXA-204 producers had a decreased susceptibility (intermediate or resistant) to ertapenem (7/15 of the *E. coli* and 11/11 of the *K. pneumonia*e isolates). All *P. mirabilis*, *C. freundii* and *S. marcescens* isolates were susceptible to imipenem and meropenem and had decreased susceptibility to ertapenem (Table 2). Regarding the broad-spectrum cephalosporins, all isolates were resistant to ceftazidime and cefotaxime. Since OXA-204 has no hydrolytic activity towards broad-spectrum cephalosporins, we searched for the expression of additional beta-lactamases (extended-spectrum beta-lactamases (ESBLs) and/or cephalosporinases). As expected, all OXA-204-producing isolates co-produced an AmpC-type beta-lactamase, CMY-4 (Table 2). In addition, 18 isolates were of intermediate susceptibility or resistant to cefepime (nine *E. coli* isolates and nine *K. pneumoniae* isolates). CMY-4 and OXA-204 are not able to hydrolyse cefepime, but co-production of the CTX-M-15 ESBL was found in all those isolates (Table 2).

### Susceptibility to non-beta-lactams antibiotics

Four antibiotics were active against the majority of the isolates. Except for the *P. mirabilis* and *S. marcescens* isolates that are intrinsically resistant to polymyxins, all other OXA-204 producers were susceptible to colistin. In addition, 28, 26 and 22 of the 29 OXA-204 producers were susceptible to amikacin, tigecycline and fosfomycin, respectively (Table 2). Conversely, 22 OXA-204 producers were resistant to sulfamethoxazole-trimethoprim, and 24 were resistant to ciprofloxacin and gentamicin (Table 2).

### Multilocus sequence typing

ST90 was the most commonly observed ST for the *E. coli* isolates, accounting for 12 of 15 isolates. The three remaining single isolates belonged to ST104, ST617 and ST949 (Table 2). Among the 11 OXA-204-positive *K. pneumoniae* isolates, nine isolates belonged to ST147. The remaining two isolates belonged to the new ST1683 and ST1709 (Table 2).

### Genetic support of the *bla*_OXA-204_ gene

Using mating-out assays, transconjugants harbouring the *bla*_OXA-204_ and the *bla*_CMY-4_ genes were obtained for all 29 strains. Plasmid DNA analysis of those transconjugants revealed a single plasmid (ca 150 kb), which was identified as an IncA/C-type plasmid (Table 2).

### Close genetic environment of the blaOXA-204 gene

The genetic environment of the *bla*_OXA-204_ gene was analysed using specific primers designed from the plasmid p204-B of *K. pneumonia*e 204 (Table 1). For 17 strains, the *bla*_OXA-204_ gene was part of transposon Tn*2016,* where IS*Ecp1* was disrupted by insertion of IS*Kpn15*. This transposon was identified in the 12 ST90 *E. coli* strains and in five other single isolates (Table 2). In the 12 remaining isolates (nine ST147 *K. pneumoniae* isolates and three single isolates), IS*Ecp1* was not disrupted by IS*Kpn15* element (Table 2).

### Endoscopy-related outbreak

**Figure 1:**
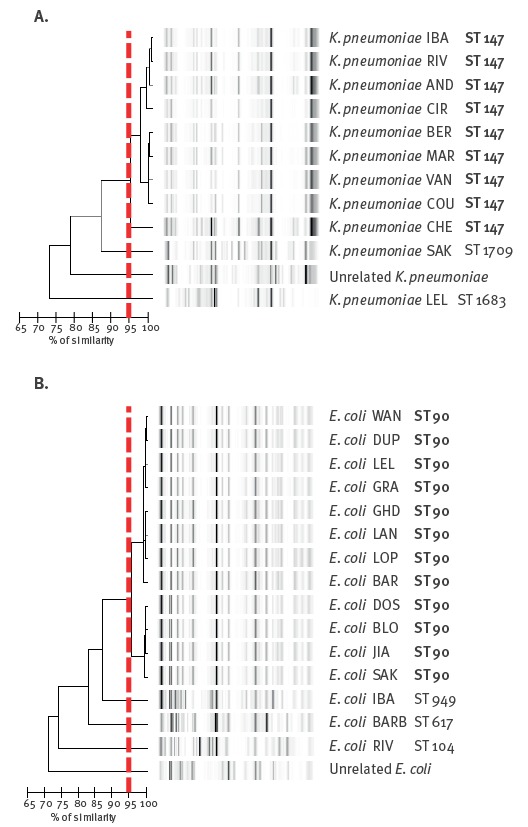
Rep-PCR analysis of OXA-204-producing *Enterobacteriaceae*, France, 2012–14 (n = 21)

Rep-PCR analysis confirmed the MLST results and showed that 12 of the 15 OXA-204-producing *E. coli* isolates (all ST90 *E. coli*) and nine of the 12 OXA-204-producing *K. pneumoniae* isolates (all ST147 *K. pneumoniae*) were clonally related (Figure 1).

Dendrogram and computer-generated image of rep-PCR banding patterns of OXA-204-producing *Klebsiella pneumoniae* (A) and OXA-204-producing *E. coli* (B) isolates. Epidemic clones (ST90 *Escherichia coli* and ST147 *K. pneumoniae* are highlighted in bold. The red similarity line at 95% shows the cut-off to separate different clone.

**Figure 2:**
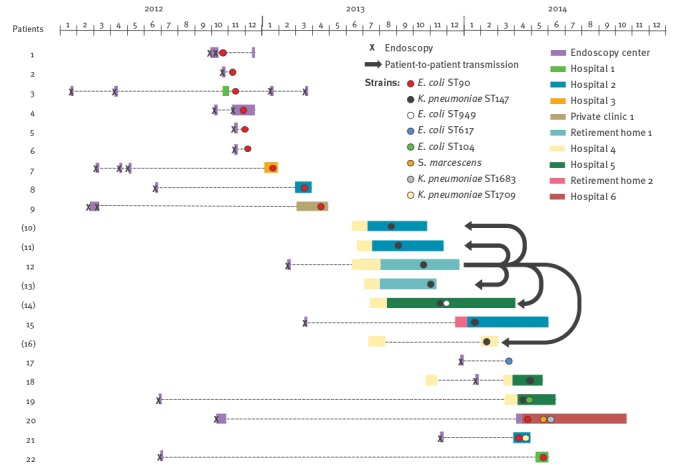
Synoptic curve of patients involved in the endoscope-related outbreak caused by OXA-204-producing *Enterobacteriaceae*, France, 2012–14 (n = 22)

The results of this analysis led us to do an epidemiologically investigation of this dual outbreak (ST90 *E. coli* and ST147 *K. pneumoniae*). An endoscope was identified as the possible source of the outbreak in that the investigation showed that 17 patients had direct contact with the endoscope, while five (Patients 10, 11, 13, 14 and 16) were considered as secondary cases through patient-to-patient transmission on a clinical ward (Figure 2).

Patient numbers are indicated on the left of each time line. Numbers in parenthesis indicate patients who were not in direct contact with the suspectedly contaminated endoscope. Crosses indicate the date of endoscopy with the suspectedly contaminated endoscope and coloured boxes correspond to hospitalisations. Black arrows represent direct patient-to-patient transmission of the OXA-204-producing *K. pneumoniae* ST147.

Of note, retrospective screening of all patients who had endoscopy with the suspectedly contaminated endoscope but were not hospitalised identified two colonised patients who underwent endoscopy as outpatients (Patients 6 and 17) (Figure 2). In addition, for four patients, transmission of a *bla*_OXA-204_-carrying plasmid from one strain to another was observed (several enterobacterial isolates of different species carrying the same plasmid with the same close genetic environment for *bla*_OXA-204_ were isolated from patients 14, 19, 20 and 21; Figure 2 and Table 2). Finally, 14 patients were infected (four biliary infections, one hepatic abscess and nine urinary tract infections) and 12 patients were colonised. For one patient (Patient 19), the acquired biliary tract infection resulted in fatal septicaemia. Overall, this outbreak spread in 10 health institutions including one endoscopy centre where patients received the endoscopy with the suspected endoscope, six hospitals, one private clinic and two retirement homes (Figure 2). 

In February 2014, the sequestration of the endoscope immediately stopped further detection of colonised patients, confirming that the endoscope was probably the source of this outbreak. However, audit of the reprocessing procedures that were performed in accordance with the manufacturer guidelines and French recommendations [25,26] did not reveal any dysfunction. In microbiological investigations of the incriminated endoscope in February 2014, no OXA-204-producing strain was recovered from the device. However, polymorphic human flora was cultured from the endoscope and three additional reprocessing procedures were needed until the device was clean enough to conform with the French recommendations [26]. 

## Discussion

We analysed different features of the OXA-204-positive enterobacterial isolates collected between October 2012 and May 2014 at the NRC for Antibiotic Resistance (division of carbapenemase-producing *Enterobacteriaceae*), France.

According to the EUCAST guidelines, 21 of the 29 OXA-204 producers remained susceptible to imipenem and meropenem, complicating their detection. Similar phenotypical characteristics have already been reported for OXA-48-producers [27]. OXA-204-producing *E. coli* seem to be more susceptible to those two carbapenems than OXA-204-producing *K. pneumoniae*. By contrast, ertapenem appeared to be the best carbapenem to detect those strains since 21 of the 29 isolates were resistant. All strains were resistant to extended-spectrum cephalosporins because of the production of the beta-lactamase CMY-4, thus limiting therapeutic options. In addition, 18 of the 29 OXA-204 producing isolates also produced a CTX-M-15-type ESBL, compromising the efficiency of cefepime. However, most of the isolates (27/29) remained susceptible to colistin, tigecycline, amikacin and fosfomycin.

We investigated the clonal distribution of OXA-204-positive isolates and identified two main STs. Twelve of the 15 *E. coli* isolates belonged to ST90. To our knowledge, this ST has not been reported to be associated with OXA-48-like-producing *E. coli*. However, the occurrence of other carbapenemases such as NDM-1 had been reported twice in ST90 *E. coli* isolates, and a link with India was demonstrated [28,29]. Nine of 11 the *K. pneumoniae* isolates belonged to ST147, which was the predominant ST in our study. The ST147 *K. pneumoniae* clone is linked to the worldwide spread of different carbapenemases (OXA-48, OXA-204, NDM-1, NDM-5, VIM-1, KPC-2) and ESBL (SHV-12, CTX-M-15) [15,27,30-33]. Additional single STs were identified, namely ST617, ST104 and ST949 for *E. coli* and two new STs (ST1683 and ST1709) for *K. pneumoniae*, supporting the hypothesis that a single *bla*_OXA-204_-positive plasmid is spreading among various genetic backgrounds (Figure 2). One OXA-204-positive ST617 *E. coli* strain was identified in Tunisia in 2015 [14]. Of note, ST617 is a widespread ST type, associated with various beta-lactamase-encoding genes (CTX-M-15, NDM-1) [34,35]. Interestingly, ESBL-producing ST617 *E. coli* were recently recovered from companion and farm animals in Tanzania and from water samples in Tunisia [36,37].

In this study, between October 2012 and May 2014, OXA-204 ST90 *E. coli* strains were regularly identified. Concomitantly, OXA-204-producing ST147 *K. pneumoniae* isolates were identified in the same area (Paris and its suburbs) between August 2013 and May 2014. Although microbiological investigations were not conclusive, our results strongly suggest that the endoscope may have been contaminated with at least two OXA-204 producing isolates: ST90 *E. coli* that co-harboured a *bla*_CTX-M-15_-carrying plasmid and ST147 *K. pneumoniae* (Figure 2). Interestingly, following this outbreak the manufacturer released several notes concerning the reprocessing procedures of the endoscope [38] and updated in June 2015 the manual for reprocessing procedures which now includes reference to a novel brush (MAJ-1888/MyBrush) [39]. This dual outbreak was controlled in May 2014 after the sequestration of the endoscope suspected to be the source of the outbreak. Gastrointestinal endoscopy has previously been identified as a risk factor for infection and colonisation with carbapenemase-producing *Enterobacteriaceae* [40]. 

Four patients were colonised or infected with more than one OXA-204-producing enterobacterial species. Three patients were colonised with one OXA-204-positive *E. coli* plus one OXA-204-producing *K. pneumoniae*, and one patient with one OXA-204-positive *E. coli* plus one OXA-204-producing *K. pneumoniae* and one additional OXA-204-positive *S. marcescens*. Those results suggest a high conjugative ability of the OXA-204-IncA/C-type plasmids. As shown for OXA-48 [41], OXA-204 is associated with an efficient genetic vehicle, thus promoting the interspecies spread of *bla*_OXA-204_-carrying plasmids among various enterobacterial species in the same patient. In addition, as previously reported, endoscopy-associated transmission of carbapenemase-producing Enterobacteriaceae (CPE) might result in long-term carriage of the acquired CPE [42] that poses a risk of further secondary outbreaks from primary infected or colonised patients. Indeed, these patients are often at high risk of recurrence of a hepato-biliary infection that needs to be treated by endoscopic procedures. To decrease the risk of secondary outbreaks, we propose making a note in these these CPE patients’ record and systematically screening them before performing any endoscopy.

The transposon Tn*2016* was identified in 17 of the 29 isolates. The truncation of IS*Ecp1* by another IS may have stabilised this genetic structure on the IncA/C plasmid by disrupting the IS*Ecp1* transposase activity. However, in the 12 remaining isolates, the IS*Ecp1* copy was intact and we can therefore speculate that the transposon made of IS*Ecp1* and *bla*_OXA-204_ is functional. IS*Ecp1* is known to be an efficient genetic vehicle for spreading clinically significant beta-lactamases such as CMY or CTX-M-15 [43,44]. The association of *bla*_OXA-204_ with an intact copy of IS*Ecp1* on the one hand and an IncA/C broad host range plasmid on the other might increase the capability of the *bla*_OXA-204_ to disseminate among various genetic elements (plasmids, chromosome, etc) and among various bacterial species.
